# Treatment outcomes of tubal pregnancy with tubal preservation: A meta-analysis

**DOI:** 10.1097/MD.0000000000036165

**Published:** 2023-11-24

**Authors:** Yan Long, Yong Lin, Jin He, Rong Zhu

**Affiliations:** a Luzhou Maternal and Child Health Hospital (Luzhou Second People’s Hospital), Sichuan Province, China.

**Keywords:** Ectopic pregnancy, intrauterine pregnancy, MTX, salpingectomy, salpingotomy, tubal pregnancy

## Abstract

**Background:**

Ectopic pregnancy is a common gynecological emergency that poses a significant risk of maternal mortality during the first trimester. It also increases the incidence of infertility and repeated ectopic pregnancy. The aim of this study was to evaluate whether there is a difference in the degree of tubal patency between salpingostomy and systemic treatment with methotrexate (MTX), as well as the odds of intrauterine pregnancy and repeat ectopic pregnancy, and the degree of tubal patency in salpingectomy with or without tubal suturing.

**Methods:**

We searched PubMed, EMBASE, and the Cochrane Library up to April 2023. Four randomized controlled trials were included in the review. We analyzed the combined data using Review Manager 5.3 software and Stata 12.0 software, utilizing a random effects model.

**Results:**

When comparing salpingostomy and systemic treatment with MTX, there was no significant difference in the degree of tubal patency (OR = 1.09, 95% CI (0.54–2.38), *P* = .83). For salpingostomy with or without tubal suturing, there were no significant differences in the rates of intrauterine pregnancy, repeat ectopic pregnancy, and tubal patency degree [(OR = 1.05, 95% CI (0.41–2.68), *P* = .92), (OR = 0.68, 95% CI (0.19–2.42), *P* = .92), (OR = 1.68, 95% CI (0.14–20.33), *P* = .68)].

**Conclusion:**

This meta-analysis demonstrates that systemic treatment with MTX is an effective treatment for patients who wish to preserve their fallopian tubes without undergoing surgery. This form of treatment can help avoid surgical procedures that may damage the fallopian tubes and improve fertility prospects. If choosing surgery, we believe that opting for salpingostomy without tubal suturing could reduce the operation time and minimize damage.

## 1. Introduction

Tubal pregnancy is the most common type of ectopic pregnancy, where a fertilized egg implants in the fallopian tube. The incidence of tubal pregnancy in women during early pregnancy is 2% to 3%.^[1]^ Furthermore, tubal pregnancy is the primary cause of maternal mortality during early pregnancy.^[2]^ Treatment options for tubal pregnancy include expectant management, medical treatment, and surgery (via laparotomy or laparoscopy). Expectant management of tubal pregnancy is also possible because early tubal pregnancy is a self-limiting disease that can be resolved naturally. Methotrexate (MTX) is the primary component used in the medical management of tubal pregnancy. Methotrexate, an antifolate acid and antitumor agent, has been identified by multiple independent lines of evidence as an inhibitor of the JAK/STAT pathway.^[3]^ Laparoscopy is the preferred method for surgically treating tubal pregnancy, with 2 surgical options: radical (salpingectomy) and conservative (salpingotomy). The treatment options for the retaining oviduct included salpingostomy or treatment with MTX, the degree of tubal patency between salpingostomy and systemic treatment with MTX, the odds of intrauterine pregnancy and repeat ectopic pregnancy, and the degree of tubal patency between salpingectomy with or without tubal suturing, no systematic review or meta-analysis has estimated these outcomes. These issues were worthy of attention and needed to be addressed. Therefore, further evaluation through a meta-analysis is needed to provide a reliable alternative for women with tubal pregnancy and clinicians.

## 2. Methods

### 2.1. Information sources and search strategy

PubMed, EMBASE, and the Cochrane Library were searched from inception to April 2023. References cited in research articles were also carefully evaluated. We used the following search terms: “ectopic pregnancy” or “tubal pregnancy,” “salpingostomy,” and “methotrexate.” The language was limited to English. In addition, we evaluated the references of the included articles and selected more relevant studies.

### 2.2. Eligibility criteria

Inclusion criteria were as follows: Only included randomized controlled trials (RCTs). Treatment options only included salpingostomy and MTX. Treatment outcome must include the degree of tubal patency.

Exclusion criteria were as follows: Animal experiments, cell studies, reviews, meta-analyses, replications, case reports, or letters were not included. Literature without outcome indicators. Studies with unusable data. Duplicate publications.

### 2.3. Data extraction and quality evaluation

Both reviewers independently judged all citations identified by the search strategy (Yong Lin and Yan Long). Abstracts of all citations were obtained to determine the eligible studies. Complete reports of all eligible studies were obtained to assess whether these studies meet the predefined inclusion criteria. Disagreements of opinion were registered and resolved by consensus among all authors. For eligible studies, we collected the relevant information, such as ectopic pregnancy characteristics (e.g., ectopic pregnancy size, mean serum human chorionic gonadotropin concentration), interventions, and outcomes. Whenever necessary, we attempted to obtain missing data by contacting the primary authors. For RCTs, we conducted a risk of bias assessment based on the criteria outlined in the Cochrane Intervention Systematic Evaluation Manual.

### 2.4. Statistical analysis

Statistical analysis was performed using Review Manager 5.3 software and Stata 12.0 software. The odds ratio (OR) and 95% confidence interval (CI) were used as effect indicators for count data analysis. The heterogeneity test includes the Cochrane *Q* test (chi-square) and I2. When I2 ≥ 50% or *P* < .1, indicating excellent heterogeneity in the study, subgroup analysis was conducted to investigate the factors contributing to non-homogeneity from both clinical and methodological perspectives. After excluding the influence of obvious clinical heterogeneity, a random-effects model was used for the analysis. Descriptive analysis was used if there was significant heterogeneity or clinical heterogeneity between the 2 groups. When I2 < 50% and *P* ≥ .1, the interstudy heterogeneity was considered small, and a fixed-effect model was selected for merging. A funnel scatter plot and Begg tests were used to determine if there was publication bias.

### 2.5. Ethics approval

No ethical approval is needed.

## 3. Results

### 3.1. Research characteristics

**Figure 1.**
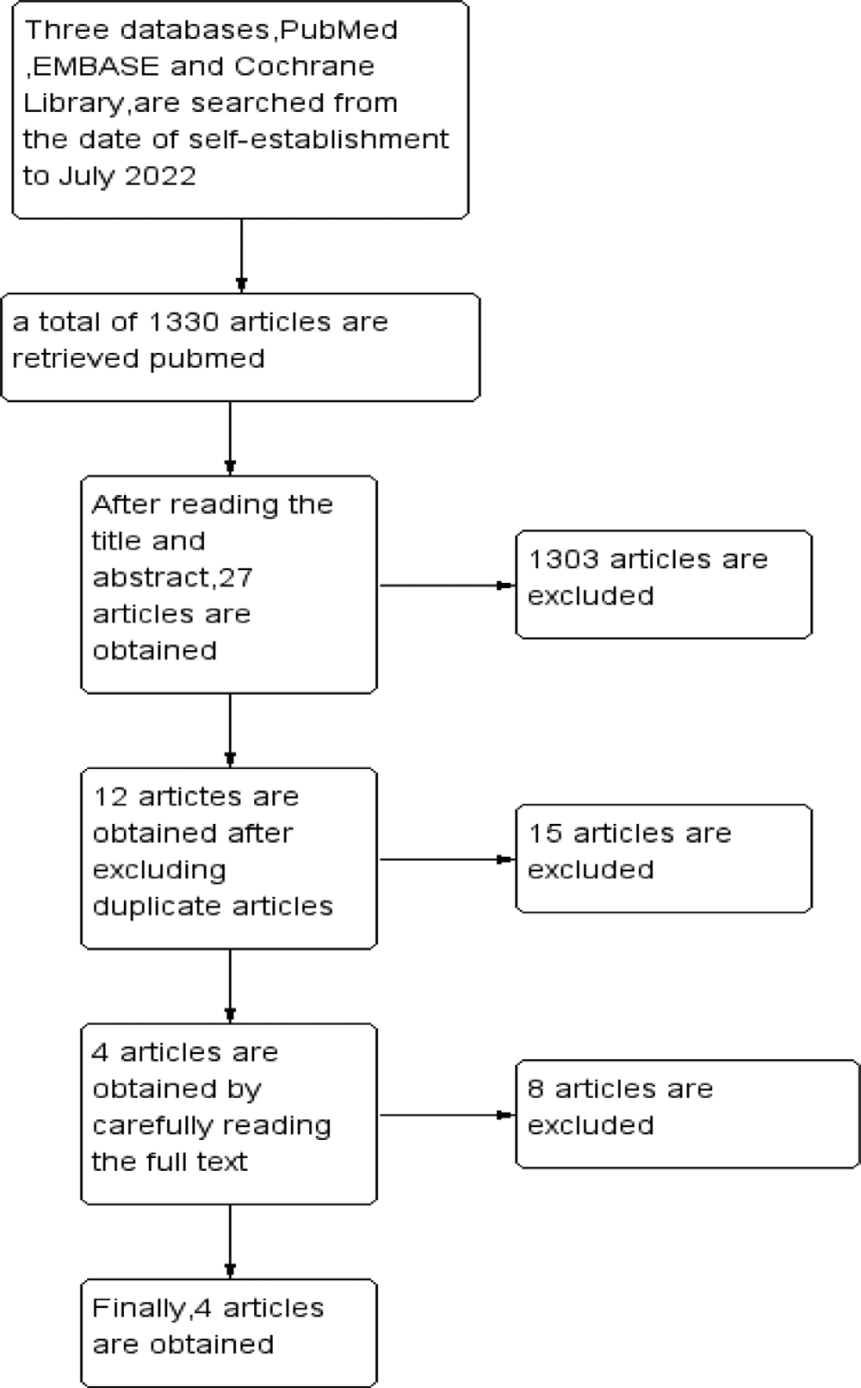
The study identification and selection process.

After excluding duplicates, 4 reports were identified using search strategies.^[4–7]^ The process of identifying and selecting the studies is illustrated in the flowchart shown in Figure 1. The characteristics of the included studies are shown in Table 1.

**Table 1:** The characteristics of the included studies.

Authors	Inclusion criteria		Intervening measure/comparison intervention	Outcome
	Pregnant bursa size	Serum HCG		
Akira Fujishita1 2004	1.5–6.0 cm	1298–2344U/L	Salpingotomy without suturing/salpingotomy with suturing	Intrauterine pregnancy, repeat ectopic egnancy, tubal patency degree
Togas Tuland i.M.D 1991	2–3 cm	Gestational age: 5.1–8 W	Salpingotomy without suturing/salpingotomy withsuturing	Intrauterine pregnancy, repeat ectopic regnancy
P J Hajenius19 97	2.0–2.3 cm	110–19500 IU/L	Salpingotomy/MTX (1 mL/kg)	Tubal patency degree
Martin C.Sowter 2001	<3.5 cm	89–4866 IU/L	Salpingotomy/MTX	Tubal patency degree

HCG = human chorionic gonadotropin.

### 3.2. Analysis of the included studies’ risk of bias

**Figure 2.**
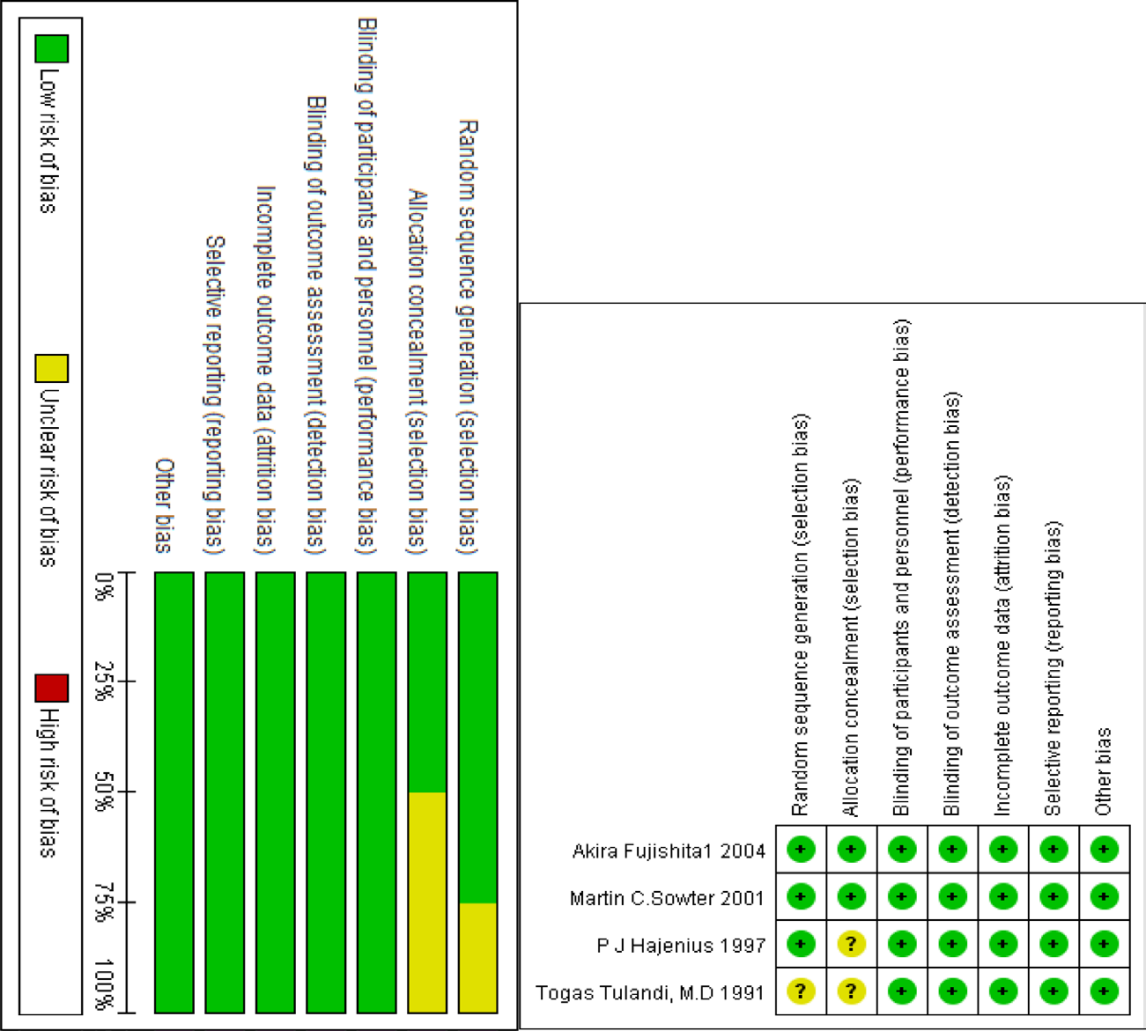
Risk-bias map.

Four of the included studies were RCTs. All trials were published in full text and have high sensitivity. Through the risk-bias map, reviewers determined that this would be unlikely to significantly influence outcome measures, which were primarily objective. The included RCTs were of high quality, as shown in Figure 2.

### 3.3. Statistical analysis results

#### 3.3.1. The degree of tubal patency after systemic treatment with MTX and salpingostomy.

**Figure 3.**
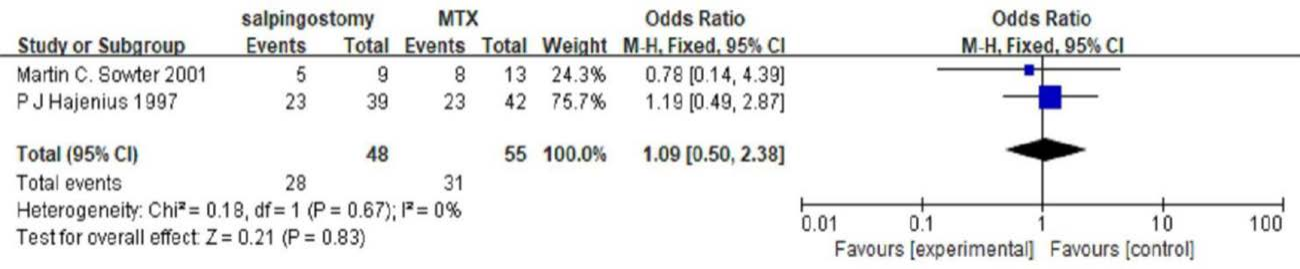
The tubal patency degree comparing systemic single-dose MTX and salpingostomy. MTX = methotrexate.

Comparing systemic treatment with MTX and salpingostomy, 2 studies were included. We used a fixed-effects model to estimate the odds ratio (OR) (Chi² = 0.18, df = 31(*P* = .67); I² = 0%). There was no significant difference in fallopian tube patency between systemic treatment with MTX and salpingostomy (OR = 1.09, 95% CI (0.54–2.38), *P* = .83) (*P* = 1.00 for Begg test) (Fig. 3).

#### 3.3.2. Salpingostomy with or without tubal suturing.

**Figure 4.**
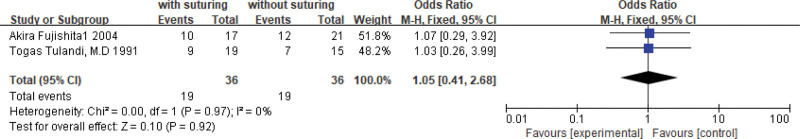
Intrauterine pregnancy comparing suturing and no suturing during salpingostomy.

**Figure 5.**
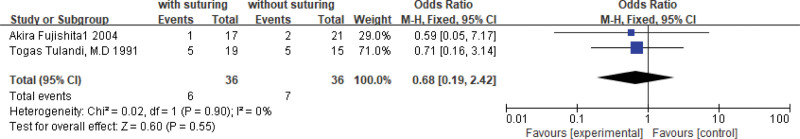
Repeat ectopic pregnancies comparing suturing and no suturing during salpingostomy.

**Figure 6.**
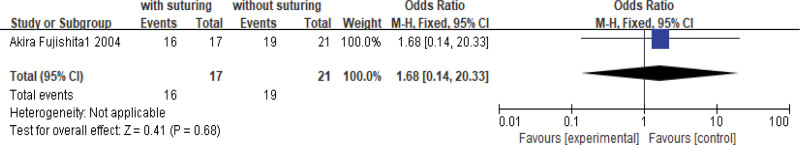
Tubal patency degree comparing suturing and no suturing during salpingostomy.

Two studies are included. In our study on salpingostomy, we compared the outcomes of suturing and non-suturing techniques. The results showed no significant difference in terms of intrauterine pregnancy (OR = 1.05, 95% CI 0.41–2.68, *P* = .92) (*P* = 1.00 for Begg test) (Fig. 4), repeat ectopic pregnancy (OR = 0.68, 95% CI 0.19–2.42, *P* = .92) (*P* = 1.00 for Begg test) (Fig. 5), and the degree of tubal patency (OR = 1.68, 95% CI 0.14–20.33, *P* = .68) (Fig. 6).

### 3.4. Sensitivity analysis

**Figure 7.**
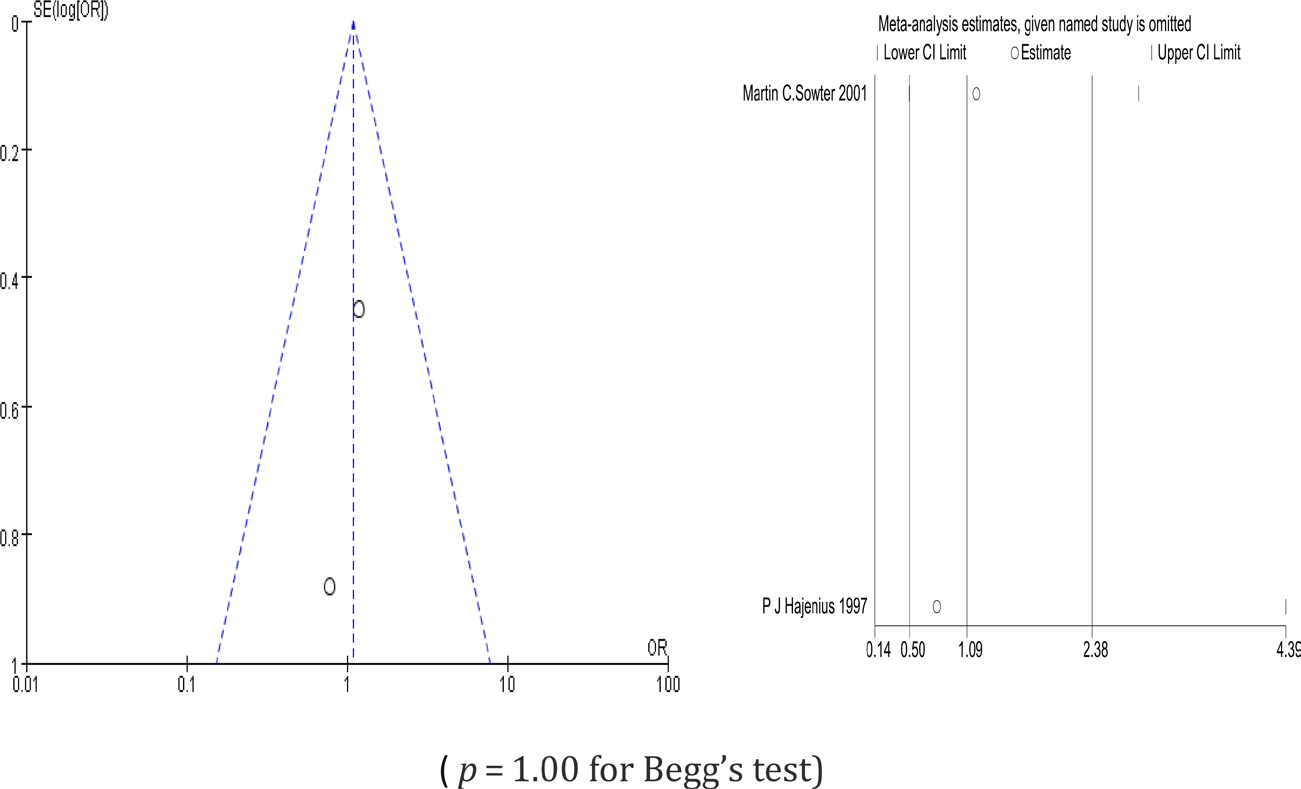
Tubal patency degree comparing methotrexate systemic treatment and salpingostomy.

**Figure 8.**
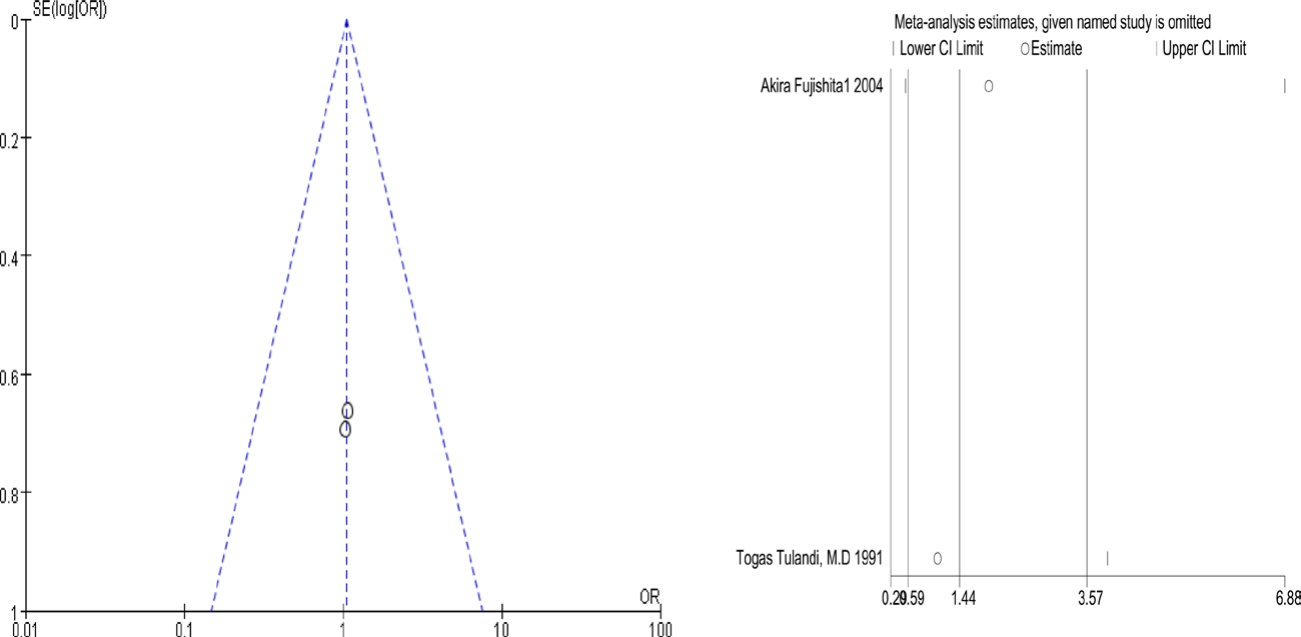
Intrauterine pregnancy comparing suturing and no suturing during salpingostomy.

**Figure 9.**
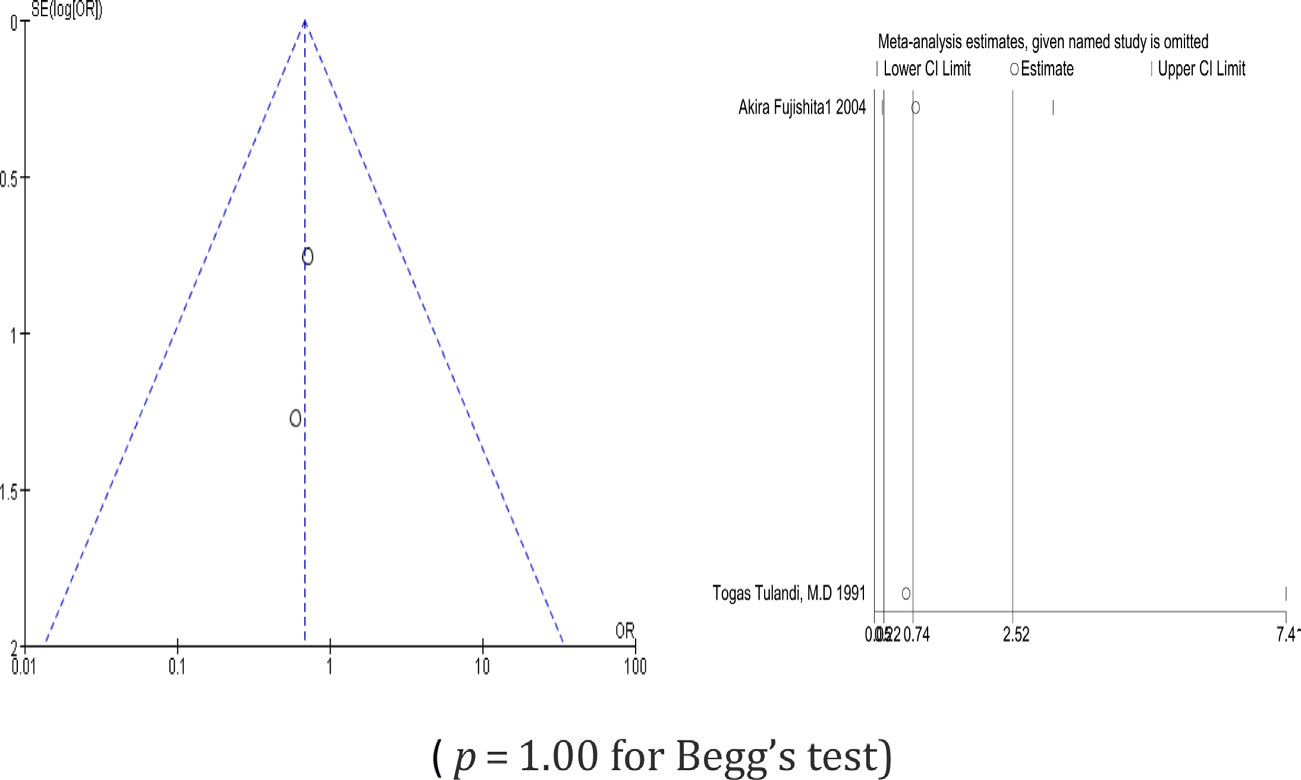
Repeat ectopic pregnancy comparing suturing and no suturing during salpingostomy.

Sensitivity analysis was conducted by omitting 1 study at a time to assess its effect on the pooled results. As indicated by the results of the analysis, all of the pooled results with 95% CIs were not remarkably influenced by any individual study. This demonstrates that the results of this meta-analysis are relatively reliable in total. The results of the sensitivity analysis are shown in Figures 7–9.

### 3.5. Publication bias

The funnel plots for the included studies were roughly symmetric (Figs. 7–9). We also conducted Begg tests to evaluate the presence of publication bias in this study. No significantly different all results emerge (Figs. 7–9).

## 4. Discussion

Hao et al showed that there was no significant difference in the odds of subsequent intrauterine pregnancy and repeat ectopic pregnancy following treatment with MTX compared to salpingostomy.^[8]^ However, no studies have shown the effect of tubal patency on a second pregnancy.

The condition of the fallopian tube after tubal pregnancy treatment, whether it is unobstructed or not, has a significant impact on future pregnancies. If the fallopian tube is obstructed, there is a possibility of another tubal pregnancy. However, if it is unobstructed, it can improve the rate of intrauterine pregnancy and preserve fertility more effectively.

This study found that there was no significant difference in the degree of tubal patency between systemic MTX and salpingostomy (OR = 1.09, 95% CI (0.54–2.38), *P* = .83). The forest plots in this study showed no obvious heterogeneity, and the funnel plots and Begg test indicated no significant publication bias. Therefore, for ectopic pregnancy with hemodynamic stability and appropriate indications, MTX treatment should be prioritized to minimize the physical trauma to the body and the fallopian tube caused by surgery. This approach aims to better preserve future fertility.^[9]^

Although systemic treatment with MTX was found to be safe and effective for treating tubal pregnancy, it did not result in cost reduction compared to salpingostomy.^[10]^ For individuals who are not suitable candidates for MTX treatment, salpingostomy is the preferred treatment option, as it may improve the chances of future pregnancies.^[11]^ This study revealed that there were no significant differences in intrauterine pregnancy, repeat ectopic pregnancy, and the degree of tubal patency between salpingostomy with or without tubal suturing [(OR = 1.05, 95% CI (0.41–2.68), *P* = .92), (OR = 0.68, 95% CI (0.19–2.42), *P* = .92), (OR = 1.68, 95% CI (0.14–20.33), *P* = .68)]. The heterogeneity analysis in this study showed low heterogeneity, and there was no obvious publication bias. Therefore, we chose salpingostomy without tubal suturing, which can reduce the operation time and minimize damage.

## 5. The strengths and weaknesses

We implemented extensive search strategies and employed effective statistical synthesis. All of the studies had a low risk of bias. The forest plot suggested no obvious heterogeneity. Additionally, the funnel plot and Begg test indicated no obvious publication bias. However, only 4 RCTs were included in this review. More high-quality RCTs are needed to improve the quality of the article. Most studies do not consider the patency of the affected fallopian tube and the contralateral fallopian tube. Future studies should consider including this information. The number of studies investigating the relationship between tubal patency and pregnancy outcomes is limited.

## 6. Conclusion

In summary, this meta-analysis demonstrates that systemic treatment with MTX is a viable option for patients who wish to preserve their fallopian tubes without undergoing surgery. This form of treatment may help avoid surgical procedures that could potentially damage the fallopian tubes and provide better prospects for fertility. If choosing surgery, we believe that opting for salpingostomy without tubal suturing could reduce the operation time and minimize damage.

## Author contributions

**Data curation:** Rong Zhu.

**Writing – original draft:** Jin He.

**Writing – review & editing:** Yan Long, Yong Lin.

## References

[1] Kirk E Papageorghiou AT Condous G. The diagnostic effectiveness of an initial transvaginal scan in detecting ectopic pregnancy. Hum Reprod. 2007;22:2824–8. 17855406 10.1093/humrep/dem283

[2] Committee on Practice Bulletins—Gynecology. ACOG Practice Bulletin No.191: Tubal ectopic pregnancy. Obstet Gynecol. 2018;131:e91–e103. 29470343 10.1097/AOG.0000000000002560

[3] Alqarni AM Zeidler MP. How does methotrexate work. Biochem Soc Trans. 2020;48:559–67. 32239204 10.1042/BST20190803

[4] Fujishita A Masuzaki H Khan KN. Laparoscopic salpingotomy for tubal pregnancy: comparison of linear salpingotomy with and without suturing. Hum Reprod. 2004;19:1195–200. 15044407 10.1093/humrep/deh196

[5] Hajenius PJ Engelsbel S Mol BW. Randomized trial of systemic methotrexate versus laparoscopic salpingostomy in tubal pregnancy. Lancet. 1997;350:774–9. 9297998 10.1016/s0140-6736(97)05487-1

[6] Sowter MC Farquhar CM Petrie KJ. A randomized trial comparing single dose systemic methotrexate and laparoscopic surgery for the treatment of unruptured tubal pregnancy. BJOG. 2001;108:192–203. 11236120 10.1111/j.1471-0528.2001.00038.x

[7] Tulandi T Guralnick M. Treatment of tubal ectopic pregnancy by salpingotomy with or without tubal suturing and salpingectomy. Fertil Steril. 1991;55:53–5. 1986973 10.1016/s0015-0282(16)54058-8

[8] Hao HJ Feng L Dong LF. Reproductive outcomes of ectopic pregnancy with conservative and surgical treatment: a systematic review and meta-analysis. Medicine (Baltim). 2023;102:e33621. 10.1097/MD.0000000000033621 PMC10145868 37115078

[9] Jianfeng N Xiaomin C Xiangna W. Clinical application of tubal reconstruction after laparoscopic tubal pregnancy operation. Zhongguo Xiu Fu Chong Jian Wai Ke Za Zhi. 2012;26:1088–90. 23057354

[10] Mol BW Hajenius PJ Engelsbel S. Treatment of tubal pregnancy in the netherlands: an economic comparison of systemic methotrexate administration and laparoscopic salpingostomy. Am J Obstet Gynecol. 1999;181:945–51. 10521759 10.1016/s0002-9378(99)70330-3

[11] Ozcan MCH Wilson JR Frishman GN. A systematic review and meta-analysis of surgical treatment of ectopic pregnancy with salpingectomy versus salpingostomy. J Minim Invasive Gynecol. 2021;28:656–67. 33198948 10.1016/j.jmig.2020.10.014

